# Draft Genome Sequences of Seven Pseudomonas fluorescens Subclade III Strains Isolated from Cystic Fibrosis Patients

**DOI:** 10.1128/genomeA.01285-14

**Published:** 2015-01-29

**Authors:** Brittan S. Scales, John R. Erb-Downward, Ian M. Huffnagle, John J. LiPuma, Gary B. Huffnagle

**Affiliations:** a Division of Pulmonary and Critical Care Medicine, Department of Internal Medicine, University of Michigan Medical School, Ann Arbor, Michigan, USA; b Department of Microbiology and Immunology, University of Michigan Medical School, Ann Arbor, Michigan, USA; c Department of Pediatrics and Communicable Diseases, University of Michigan Medical School, Ann Arbor, Michigan, USA

## Abstract

We report here the first draft genome sequences of Pseudomonas fluorescens strains that have been isolated from humans. The seven assembled draft genomes contained an average of 60.1% G+C content, were an average genomic size of 6.3 Mbp, and mapped by multilocus sequence analysis to subclade III.

## GENOME ANNOUNCEMENT

Cystic fibrosis (CF) is an inherited genetic disorder that affects more than 70,000 children and adults worldwide (1, 2). The mutations that cause CF lead to the buildup of thick mucus secretions in the lungs and defective airway clearance mechanisms, resulting in an increased microbial burden within the respiratory tract (3, 4). A complex respiratory microbiota, whose membership remains in almost constant flux, forms in individuals with CF (5–7). Of the Pseudomonas species, Pseudomonas aeruginosa is the most common species found in the CF lung, often at very high levels (2, 8–10). Though much less frequently reported, Pseudomonas fluorescens can also be detected in the sputum of individuals with CF, as well as in those with other respiratory diseases (11–13). Unlike P. aeruginosa, P. fluorescens is largely considered a resident of the plant and rhizosphere environment (12, 14). Little is known about the effects of P. fluorescens colonization on the lungs of CF patients.

Here, we report the first genome sequences of P. fluorescens strains that that have been isolated from human lungs. The strains were isolated from the sputum of seven different individuals with CF. These seven strains map by multilocus sequence analysis to P. fluorescens subclade III, for which only environmental (soil, plant rhizosphere, or plant phyllosphere) isolates have been previously reported and sequenced (15). Other members of the P. fluorescens subclade III are P. fluorescens strains SBW25, A506, WH6, and SS101 and Pseudomonas synxantha BG33R1 (15).

Isolation of the P. fluorescens strains occurred between March 2001 and January 2008 from five different treatment centers across the United States (Hartord, CT; Seattle, WA; Salt Lake City, UT; Little Rock, AR; and Augusta, GA). Strains were isolated and banked at −80°C. PCR for the 16S rRNA gene using the universal primer set 8F and 1492R (16) amplified a fragment which was sequenced by Sanger sequencing using an ABI 3730XL sequencer. The sequences were then identified as strains of P. fluorescens through a BLAST search of the amplified 16s rRNA gene. The isolates were then grown aerobically overnight in Luria broth at 34°C. Genomic DNA was isolated with the Qiagen DNeasy blood and tissue kit (catalog number 69506). Sequence data were generated with a 100-bp paired-end library on the Illumina HiSeq 2000 platform. The Illumina reads were assembled *de novo* using the DNAstar SeqMan NGen Version 12 software. The genomes were assembled into an average of 58 contigs (range 23 to 109). Contigs were ordered with the Mauve aligner (17), using a previously sequenced P. fluorescens subclade 3 genome as a reference. The assembled draft genomes contained an average of 60.1% G+C content (range, 59.5% to 60.8%) and were of an average genomic size of 6.3 Mbp (range, 6.1 to 6.8 Mbp). Multilocus sequence analysis was performed using DnaE, PpsA, RecA, RpoB, GuaA, MutL, PyrC, and AcsA, modified from the approach previously reported by Loper et al. (15). The clustering and phylogenetic tree were created using MAFFT (18, 19).

### Nucleotide sequence accession numbers.

The draft genomes have been deposited at DDBJ/EMBL/GenBank with the accession numbers JRXT00000000, JRXU00000000, JRXV00000000, JRXW00000000, JRXX00000000, JRYA00000000, and JRXY00000000 for isolates AU2989, AU6026, AU10973, AU11518, AU14440, AU14705, and AU14917, respectively.
